# Occurrence of *Vibrio* spp. along the Algerian Mediterranean coast in wild and farmed *Sparus aurata* and *Dicentrarchus labrax*

**DOI:** 10.14202/vetworld.2020.1199-1208

**Published:** 2020-06-26

**Authors:** Sonia Arab, Luca Nalbone, Filippo Giarratana, Ali Berbar

**Affiliations:** 1 Institut des Sciences Vétérinaires, Laboratoire de Recherche de Biotechnologies liées à la Reproduction Animale, University of Saad Dahlab - Blida, route de Soumâa BP 270, Blida, 09000, Algeria; 2 Department of Veterinary Sciences, University of Messina, Polo Universitario dell’Annunziata, 98168 Messina, Italy

**Keywords:** Algeria, gilt head sea bream, Mediterranean sea, sea bass, *Vibrio* spp

## Abstract

**Background and Aim:**

*Vibrio* bacteria are autochthonous inhabitants of aquatic and marine environments. Certain strains are responsible for important seafood-borne outbreaks in developed nations. The aim of this study was to investigate the occurrence of *Vibrio* spp. along the Algerian Mediterranean coast in several samples of two prominent wild and farmed marine fishes, *Sparus aurata* and *Dicentrarchus labrax*.

**Materials and Methods:**

A total of 350 *S. aurata* (n=280 farmed and n=70 wild) and 340 *D. labrax* (n=250 farmed and n=90 wild) were sampled among three different locations along the Algerian Mediterranean coastal area. The samples were processed for *Vibrio* research according to the ISO methods. Isolated colonies were identified utilizing biochemical tests and consecutively confirmed with matrix-assisted laser desorption ionization-time-of-flight mass spectrometry, combined with polymerase chain reaction (PCR) analysis when appropriate, or confirmed with PCR analysis alone.

**Results:**

A total of 42 *Vibrio* spp. were detected only among the farmed fishes. Taking into account, all 690 fishes sampled, the incidence of *Vibrio* spp. was 6.08% (with peaks up to 7.92%) among the total number of farmed fishes. Overall, 25 strains were isolated from *S. aurata* and 17 strains were isolated from *D. labrax*. The isolated strains belonged to four different species and were represented as follows: *Vibrio alginolyticus* (n=20), *Vibrio cholerae* (n=15), *Vibrio fluvialis* (n=5), and *Vibrio hollisae* (n=2). The incidence of *Vibrio* was higher in places characterized by greater levels of anthropogenic contamination of seawater.

**Conclusion:**

Considering the growing production and consequent rising consumption of farmed fish in Algeria, the reported incidence of *Vibrio* and the presence of potentially pathogenic strains of *Vibrio* such as *V. cholerae* cause particular concern for food safety matters. Even if innovative and natural techniques are desired in aquaculture, proper hygiene and manufacturing practices are essential for the correct management of *Vibrio* infection risk in farmed fishes at both industrial and domestic levels.

## Introduction

Bacteria comprising the genus *Vibrio* are known to be autochthonous inhabitants of aquatic and marine environments, as well as waterborne bacterial pathogens [1-3]. Of the genus *Vibrio*, the most widely studied species is *Vibrio cholerae*, the etiological agent of cholera [4,5]. However, in recent years, more attention has been paid to other potentially enteropathogenic *Vibrio* spp. such as *Vibrio vulnificus* and *Vibrio parahaemolyticus* as the strains in question have emerged as the predominant etiological agents of human seafood-borne infections in developed nations [4,6-12]. To date, there are more than 100 individual species in the genus *Vibrio*, but only approximately a dozen have been associated with human illness [4]. Among these pathogenic strains, *Vibrio alginolyticus*, *V. cholerae*, *Vibrio costicola*, *Vibrio mimicus*, *Vibrio cincinnatiensis*, *Vibrio hollisae*, *Vibrio fluvialis*, *Vibrio furnissii*, *V. parahaemolyticus*, *V. vulnificus*, *Vibrio* *carchariae* (a junior synonym of *Vibrio harveyi*), and *Vibrio metschnikovii* are clinically important as they are capable of causing several types of vibriosis related to the ingestion of contaminated water and undercooked seafood [4,8,13-18]. The pathogenicity of *Vibrio* is reported not only within humans but also within several aquatic organisms [19-22]. In fact, *Vibrio aguillarum* and *V. alginolyticus* are found present as pathogens in several fishes and shellfishes [16,21,22]. *V. alginolyticus* is responsible for many epizootic outbreaks in the two most important fish of Mediterranean aquaculture such as gilthead sea bream (*Sparus aurata*) and sea bass (*Dicentrarchus labrax*) [19,20,23].

The incidence and occurrence of *Vibrio* infections in humans are on the rise worldwide [4,6,7,17,24,25]. This worldwide spread of *Vibrio* is most likely related to several factors such as (i) climate change variations [11,12,26,27]; (ii) new oceanic patterns that introduce warmer waters into colder regions and change the salinity profile of coastal rivers [11,12,26,28]; (iii) the rising consumption of seafood products, especially raw or undercooked seafood [6,14,29-32]; and (iv) an increase in nutrients such as nitrogen and carbon stemming from coastal anthropization and the presence of fish farms and bathing facilities [18,31,33,34].

As reported above, considering the widespread and growing economic importance of marine fish farming for Algeria (a projection of 100,000 tons of seafood a year by 2020) [35,36] and the lack of data on the presence of pathogenic *Vibrio* for the country in question, which has been thoroughly described in wild and farmed fishes [20,37-40]. The aim of this study was to investigate the occurrence of *Vibrio* spp. along the Algerian Mediterranean coast in the two most important cultured and wild marine fish in Algeria, *S. aurata* and *D. labrax*.

## Materials and Methods

### Ethical approval

This study does not contain any experimental studies with animals. All fishes used were just died before sampling.

### Fishes collection

**Figure-1:**
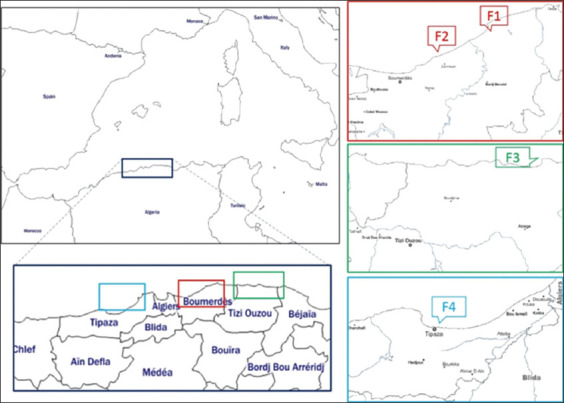
Localization of sampling area along the Mediterranean Algerian coast. In red, the Boumerdes with two fish farms F1 and F2; in green, the Azefoune area with the fish farm F3; in blue, the Tipaza area with fish farm F4.

The present survey was conducted monthly for 2 consecutive years (from January 2017 to December 2018), collecting a total of 690 wild and cultured sea bass (*D. labrax*) and gilt head sea bream (*S. aurata*) along the coast of the Algerian Mediterranean (Tables-1 and 2). Three different locations (Boumerdes, Azefoune, and Tipaza) were selected in relation to the proximity of fish farms and commercial fishing activity in the vicinity (Figure-1).

**Table-1:** Distribution of wild and farmed *Sparus aurata* collected from January 2017 to December 2018.

Area	Year	n. *Sparus aurata*												

		January	February	March	April	May	June	July	August	September	October	November	December	TOT
Boumerdes														
F1	2017	4	4	4	4	4	4	4	4	4	4	4	4	48
	2018	4	4	5	4	4	5	4	4	5	4	4	5	52
F2	2017	3	3	4	3	3	4	3	3	4	3	3	4	40
	2018	3	3	4	3	3	4	3	3	4	3	3	4	40
W	2017	2	2	1	2	2	1	2	2	1	2	2	1	20
	2018	2	2	1	2	2	1	2	2	1	2	2	1	20
Azefoune														
F3	2017	1	1	1	1	1	1	1	1	1	1	1	1	12
	2018	1	1	0	1	1	0	1	1	0	1	1	0	8
W	2017	1	0	0	1	0	0	1	1	0	1	1	0	6
	2018	1	0	0	1	0	0	1	0	0	1	0	0	4
Tipaza														
F4	2017	3	3	4	3	3	4	3	3	4	3	3	4	40
	2018	3	3	4	3	3	4	3	3	4	3	3	4	40
W	2017	1	1	1	1	1	1	1	1	1	1	1	1	12
	2018	1	1	0	1	1	0	1	1	0	1	1	0	8
TOT		30	28	29	30	28	29	30	29	29	30	29	29	350

**Table-2:** Distribution of wild and farmed *Dicentrarchus labrax* collected from January 2017 to December 2018.

Area	Year	*n. Dicentrarchus labrax*												

		January	February	March	April	May	June	July	August	September	October	November	December	TOT
Boumerdes														
F1	2017	7	7	6	7	7	6	7	7	6	7	7	6	80
	2018	7	7	6	7	7	6	7	7	6	7	7	6	80
F2	2017	0	0	0	0	0	0	0	0	0	0	0	0	0
	2018	0	0	0	0	0	0	0	0	0	0	0	0	0
W	2017	2	2	1	2	2	1	2	2	1	2	2	1	20
	2018	2	2	1	2	2	1	2	2	1	2	2	1	20
Azefoune														
F3	2017	1	1	1	1	1	1	1	1	1	1	1	1	12
	2018	1	1	0	1	1	0	1	1	0	1	1	0	8
W	2017	1	0	0	1	0	0	1	1	0	1	1	0	6
	2018	1	0	0	1	0	0	1	0	0	1	0	0	4
Tipaza														
F4	2017	3	3	2	3	3	2	3	3	3	3	3	3	34
	2018	3	3	3	3	3	3	3	3	3	3	3	3	36
W	2017	2	2	1	2	2	1	2	2	1	2	2	1	20
	2018	2	2	1	2	2	1	2	2	1	2	2	1	20
TOT		23	32	30	22	32	30	22	32	31	23	32	31	350

All the fishes examined in this study were selected among fishes with commercial dimensions (weighing at least 300 g). Farmed fishes, immediately after being harvested, were killed by immersion in an ice-water slurry.

All wild fishes, which were fished close to the relative area, were harvested directly from local fishermen within 4-6 h after their catch period.

In the Boumerdes area, a total of 420 fishes were collected: 340 cultured fishes from the two fish farms (F1 and F2) were present and 80 wild fishes were also present (40 sea bream and 40 sea bass). Regarding cultured fishes, 260 fishes (100 *S. aurata* and 160 *D. labrax*) were sampled from F1 (Cap Djinet, Algeria), while the remaining 80 fishes (only *S. aurata*) were sampled from F2 (Zemmouri, Algeria). Farm F1 was a land-based fish farm that takes in the waters of Isser Wadi alongside absorbing the hot water discharged by the Cap Djinet thermal power plant; farm F2 was, in contrast, an offshore plant. The untreated sewage discharge from the town of Zemmouri flows into the seawater of the Boumerdes area.

In the Azefoune area, a total of 60 fishes were collected: 40 cultured fishes (20 sea bream and 20 sea bass) were harvested from the fish farm F3 and 20 wild fishes (10 sea bream and 10 sea bass). Farm F3 was an offshore aquaculture area (F3), placed in a location close to Irzer M’lata Wadi.

Finally, the remaining 210 fishes were harvested from the Tipaza area: 150 cultured fishes (80 sea bream and 70 sea bass) were collected from the offshore fish farm F4 and 60 wild fishes (20 sea bream and 40 sea bass) were also collected. This area absorbed the untreated wastewater discharges from the industrial group of the town of Ain Tagourait.

### Bacteriological analysis

Once they were successfully collected, all fishes were transported to the laboratory of the Institute of Veterinary Sciences of the University of Saad Dahlab (Blinda, Algeria) in single, clean plastic containers under refrigerated condition (2-4°C) and immediately analyzed. Materials were harvested from each fish: Skin, gills, and intestinal content were aseptically all removed and rendered into small pieces utilizing sterile forceps and scalpels. The obtained mixture was divided into two aliquots: The first aliquot for the detection of *V. parahaemolyticus* and *V. cholerae* according to ISO/TS 21872-1: 2007 [41] and the second aliquot for the detection of other *Vibrio* species according to ISO/TS 21872-2: 2007 [42].

### Detection of V. parahaemolyticus and V. cholerae

Alkaline saline peptone water (ASPW) (2% of NaCl with a final pH of 8.6±0.2) at ambient temperature was combined with a ratio of 1:9 (w/v) with the obtained mixture of fish skin, gills, and intestinal content (from 10 to 25 g) and homogenized for 60 s at 230 rpm utilizing a stomacher. After an incubation period at 41.5°C for 6±1 h, 1 ml from this first enrichment in ASPW was inoculated into a tube containing 10 ml of ASPW (second enrichment – incubation at 41.5°C for 18±1 h) and simultaneously streaked with a sampling loop on the surface of thiosulfate citrate bile sucrose agar (TCBS – with 1% NaCl and incubated at 37°C per 24±1 h) (bioMerieux, Marcy l’Etoile, France). The second enrichment was also streaked with a sampling loop on TCBS and incubated in the same fashion.

From the two TCBS plates, at least five colonies of *Vibrio* considered to be typical or similar to *V. cholerae* (smooth, green, and with a diameter of 2-3 mm) and to *V. parahaemolyticus* (smooth, yellow, and with a diameter of 2-3 mm) were chosen and plated on saline nutrient agar (SNA with 1% NaCl incubated at 37°C for 24±3 h) (bioMerieux, Marcy l’Etoile, France).

### Detection of other Vibrio species

With respect to the process previously described according to ISO/TS 21872-2: 2007 [42], for the detection of other *Vibrio* species, it was necessary to alter only the temperature of incubation of the second aliquot in the two required enrichments in ASPW. In particular, the first enrichment necessitated an incubation at 37°C for 6±1 h, while the second aliquot was incubated at 37°C for 18±1 h.

From the two TCBS plates, at least five colonies considered to be typical (smooth, green, or yellow and with a diameter of 2-3 mm) were chosen and plated on SNA with 1% NaCl incubated at 37°C for 24±3 h.

### Biochemical identification

All presumptive colonies isolated on SNA underwent an oxidase test (bioMerieux, Marcy l’Etoile, France) as well as morphology, mobility, and Gram reactions utilizing microscopes. Only the oxidase-positive and Gram-negative colonies which yielded positive results in the motility test were then tested for the following biochemical confirmations: Arginine dihydrolase (bioMerieux, Marcy l’Etoile, France), L-lysine decarboxylase (bioMerieux, Marcy l’Etoile, France), and ornithine decarboxylase (bioMerieux, Marcy l’Etoile, France) and then underwent evaluation of growth in peptone water with increasing salt (NaCl) concentrations (0%, 2%, 4%, 6%, 8%, and 10%) incubated at 37°C for 24±3 h. The remaining colonies required biochemical confirmations such as detection of β-galactosidase, of indole, and tests with saline TSI agar were instead performed with an API 20E commercial kit (bioMerieux, Marcy l’Etoile, France). The confirmation of *Vibrio* species identification obtained according to ISO/TS 21872-1: 2007 [41] and ISO/TS 21872-2: 2007 [42] as well as of unreported species was finally performed according to the Noguerola and Blanch biochemical identification key [43].

### Confirm of identification

The obtained identifications utilizing biochemical tests were confirmed with matrix-assisted laser desorption ionization-time-of-flight mass spectrometry (MALDI-TOF MS) alone, a mix of MS and polymerase chain reaction (PCR) when necessary or PCR alone. These analyses were performed in the Food Inspection Laboratory of the Department of Veterinary Sciences at the University of Messina, Italy. Once the strains arrived at the laboratory, they were plated on Nutrient Agar (Biolife, Milan, Italy) or on SNA (with 2% NaCl) for non-halophilic and halophilic *Vibrio*, respectively, and incubated at 37°C for 24 h.

In addition, all the *Vibrio* strains were stored at −80°C in Brain Heart Infusion (Biolife, Milan, Italy) plus 15% of glycerol (Biolife, Milan, Italy) supplemented with 1% NaCl.

### MALDI-TOF MS identification

For MALDI-TOF MS identification, *Vibrio* colonies cultured on NA or on SNA (with 2% NaCl) were directly transferred utilizing a loopful (1 ml loop) on FlexiMass MALDI target plates, with 48-well sample spots (bioMerieux, Firenze, Italy). Each colony was smeared into a thin film on a single well, overlaid with 1 μl of matrix solution (CHCA, saturated solution of alpha-cyano-4-hydroxycinnamic acid in 500 mL/L acetonitrile, and 25 mL/L trifluoroacetic acid) and air-dried for 5 min at room temperature. *Escherichia coli* ATCC 8739, utilized as calibrator and internal ID control, and grown in blood agar (50 mL/L sheep blood) (Biolife, Italy) for 24 h, was inoculated on distinct and separate calibration spots (G3 and G4 position). After crystallization of the matrix and microbial material, the plates were put into a Vitek MS Axima Assurance mass spectrometer (bioMerieux, Firenze, Italy) in positive linear mode at a laser frequency of 50 Hz with an acceleration voltage of 20 kV with an extraction delay time of 200 ns. Mass spectra detected ranged from 2000 to 20,000 Da.

MALDI-TOF generated unique MS spectra for microorganisms that were transferred into the SARAMIS software (Spectral ARchive and Microbial Identification System – Database version V4.12 – Software year 2013, bioMerieux, Firenze, Italy) where they were compared to the database hosting the reference spectra and SuperSpectra of bacteria. The SARAMIS database contains over 25,000 spectra from 586 bacterial and fungal species [44].

Each strain was analyzed 3 times in distinct, separate runs. A percent probability, or confidence level, was calculated by the software algorithm. This value represents specific peaks between the generated spectrum and the database spectra of their similarity in terms of presence or absence. A perfect match between the sample spectrum and the unique spectrum of a single organism or bacterial group resulted in a confidence level of 99.9% (“excellent ID”). For confidence levels ranging from >60% to 99.8%, the identification was considered a “good ID” because the spectrum was adequately close to that of a reference spectrum, while for a value of <60%, “no identification” (no ID) was given [45].

### PCR identification

All isolated *Vibrio* strains were subcultured from glycerol stock onto SNA (supplemented with 1% NaCl) and incubated at 37°C for 18±1 h. According to UNI EN ISO 21872-1:2017, one well-isolated colony from each SNA plate utilizing an inoculating loop was suspended in 500 μl of sterile saline solution (0.85% NaCl) in a 1.5 ml microcentrifuge tube. DNA extraction was implemented with a heat treatment in Thermomixer (Eppendorf, Hamburg, Germany) registered at 95°C for 5 min. After this treatment, the tubes were centrifugated at 10,000 rpm for 1 min, and the supernatant was removed for PCR testing and stored at −20°C until use. DNA concentration was estimated spectrophotometrically (SmartSpec Plus, Bio-Rad, Milan, Italy).

All biochemically identified strains were identified to proper genera through amplification utilizing PCR of the gene *rpoA* that codifies for the RNA polymerase alpha subunit, as mentioned by Dalmasso *et al*. [46]. The PCR reactions were conducted in a 50 μL volume consisting of 0.5 μg of purified genomic DNA, 25 pmol of each primer, 1x PCR buffer, 2 mM MgCl_2_, 0.2 mM each of the dNTPs (Invitrogen, Paisley, UK), and 1 U of recombinant *Taq* DNA polymerase (Invitrogen, Paisley, UK).

The confirmations of *V. cholerae* were performed according to UNI EN ISO 21872-1:2017 [47] by the detection of the *prVC* target region [48]. DNA amplification was performed in a thermocycler (C1000 Touch, Bio-Rad, Italy) utilizing an initial denaturation step at 96°C for 5 min followed by 30 cycles of amplification (denaturation at 94°C for 1 min, annealing at 63°C for 1.5 min, and extension at 72°C for 1.5 min), ending with a final extension at 72°C for 7 min.

Five microliters of each amplicon were loaded into 2% agarose gel (Sigma-Aldrich, Italy) and subjected to linear electrophoresis at 90 V for 40 min. Gels were imaged using a UV transilluminator (Gel Doc XR, Bio-Rad Laboratories, Hercules, USA) and analyzed utilizing Quantity One software (Bio-Rad Laboratories, Hercules, USA). A 100 bp DNA Ladder (Invitrogen Ltd., Paisley, UK) was utilized as a reference marker. For all PCR applications, a PCR mixture without DNA was used as a negative control and the reference strains *Vibrio alginolyticus* ATCC 17749 (for *rpoA*) and *V. cholerae* CCUG 3751 (for *prVC*) were used as positive controls. All primers were synthesized by Operon Technology (Cologne, Germany). The sequences of the primers used for amplification are listed in Table-3 [46,48].

**Table-3:** PCR primer used in the study.

Gene target	Primer	Sequence	References
*rpoA*	rpoAf	Aaatcaggctcgggcct	[46]
	rpoAr	gcaatttt(a/g)tc(a/g/t)ac(c/t)gg	
*prVC*	prVCf	ttaagcsttttcrctgagaatg	[48]
	prVCr	agtcacttaaccatacaacccg	

PCR=Polymerase chain reaction

### Statistical analysis

To evaluate significant differences among the samples in question, including fishery zones, species, and commercialized variants, one-way ANOVA and T-tests were carried out (Addinsoft, Microsoft Excel). The significance level was established as p<0.05.

## Results and Discussion

**Figure-2:**
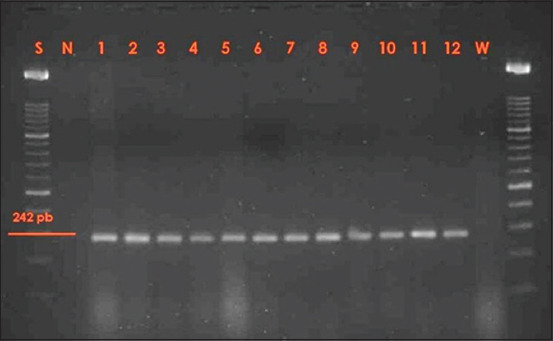
Agarose gel electrophoresis of the *rpoA* (242 pb) amplification products of *Vibrio* strains. Lane S: Scale with 100 bp molecular size marker; lane N: Negative control (polymerase chain reaction mixture without DNA); lane 1: Positive control (*Vibrio alginolyticus* ATCC 17749); lanes 2-12: Positive samples; lane W: Water control.

The application of the two ISO methods [41,42] permitted us to identify 42 strains as *Vibrio spp*. and to identify 15 strains as *V. cholerae* and 5 as *V. fluvialis*. The Noguerola and Blanch key [43] allowed our team to identify the remaining 22 strains (n=20 *Vibrio alginolyticus* and n=2 *V. hollisae*) and to confirm their previous identification. The following MALDI-TOF analysis and PCR (*rpoA* and *prVC*) tests validated the achieved results (Figure-2). All the isolated strains of *V. cholerae* were no-O1 and no-O139 and yielded negative results to the agglutination test with specific antisera.

Among 690 fishes analyzed, the 42 *Vibrio* strains were detected only in the cultured samples (from 42 different fishes) (Tables-4 and 5). No *Vibrio* spp. were, in fact, detected among wild fishes. The farm setting may determine alterations in the chemical-physical parameters of the seawater such as to favor the cultivation of certain bacterial strains, including pathogens [1,49]. In addition, exposure to pathogens may be higher and more expansive in a farmed aquaculture setup than among wild fishes. Overall, 25 and 17 strains were isolated from cultured *S. aurata* and cultured *D. labrax*, respectively, without any significant difference (p>0.05) (Table-4). In both species, *V. alginolyticus*, *V. cholerae*, and *V. fluvialis* were identified, while the two strains of *V. hollisae* were detected only in *D. labrax*.

**Table-4:** Distribution of *Vibrio* strains isolated in farmed *D. labrax* and *S. aurata* in relation to the season.

Strain	Season	*S. aurata*				*D. labrax*				Total
	
		F1	F2	F3	F4	F1	F2	F3	F4	
*V. alginolyticus*	Winter	1	0	0	0	1	0	0	0	2
	Spring	0	0	0	0	0	0	0	0	0
	Summer	3	4	0	2	2	0	0	2	13
	Autumn	1	1	0	1	1	0	0	1	5
*V. cholerae*	Winter	1	0	0	0	1	0	0	0	2
	Spring	0	0	0	0	0	0	0	0	2
	Summer	2	4	0	1	2	0	0	1	0
	Autumn	0	0	0	2	0	0	0	1	10
*V. fluvialis*	Winter	0	0	0	0	0	0	0	0	3
	Spring	0	0	0	0	0	0	0	0	0
	Summer	0	0	0	1	0	0	0	2	0
	Autumn	0	0	0	1	0	0	0	1	3
*V. hollisae*	Winter	0	0	0	0	0	0	0	0	0
	Spring	0	0	0	0	0	0	0	0	0
	Summer	0	0	0	0	2	0	0	0	2
	Autumn	0	0	0	0	0	0	0	0	0
	Total		8	9	8	9	0	0	42

S. aurata=Sparus aurata, D. labrax=Dicentrarchus labrax, V. alginolyticus=V. alginolyticus, V. cholerae=Vibrio cholerae, V. fluvialis=Vibrio fluvialis, V. hollisae=Vibrio hollisae

**Table-5:** Distribution of the different *Vibrio* strains isolated in farmed *D. labrax* and *S. aurata* from January 2017 to December 2018.

Sample	Year	Area	January	February	March	April	May	June	July	August	September	October	November	December
*S. aurata*	2017	F1	-	1 *Vc*	-	-	-	-	-	2 *Vc* 3 *Va*	-	-	1 *Va*	-
		F2	-	-	-	-	-	-	1 *Vc* 1 *Va*	1 *Vc* 1 *Va*	-	-	1 *Va*	-
		F4	-	-	-	-	-	-	-	1 *Vc* 1 *Va*	-	1 *Vc* 1 *Va*	-	-
	2018	F1	-	1 *Va*	-	-	-	-	-	-	-	-	-	-
		F2	-	-	-	-	-	-	1 *Vc* 1 *Va*	1 *Vc* 1 *Va*	-	-	-	-
		F4	-	-	-					1 *Va* 1 *Vf*	-	1 *Vc* 1 *Vf*	-	-
*D. labrax*	2017	F1	-	1 *Vc* 1 *Va*	-	-	-	-	-	1 *Vc* 1 *Va* 2*Vh*	-	1 *Va*	-	-
		F2	-	-	-	-	-	-	-		-		-	-
		F4	-	-	-	-	-	-	-	1 *Va*	-	1 *Va*	-	-
	2018	F1	-	-	-	-	-	-	-	1 *Vc* 1 *Va*	-	-	-	-
		F2	-	-	-	-	-	-	-		-	-	-	-
		F4	-	-	-	-	-	-	-	1 *Vc* 1 *Va* 2*Vf*	-	1 *Vc* 1 *Vf*	-	-

Vc=V. cholera, Va=V. alginolyticus, Vf=V. fluvialis, Vh=V. hollisae, S. aurata=Sparus aurata, D. labrax=Dicentrarchus labrax

The isolation of *V. hollisae* in fishes is uncommon due to the stringent growth conditions that cause typically low growth of these strains on selective media for *Vibrio* spp. such as those used in this study [13]. Among these 42 strains, *V. alginolyticus* was the most representative species (48%), followed by *V. cholerae* (36%), *V. fluvialis* (12%), and *V. hollisae* (4%).

The recovery of *Vibrio* spp. was affected by the season in which they were sampled (Table-4). Among all the fishes, the largest amount (n=28) of strains was detected during the summer and principally in August (Table-5), while no *Vibrio* spp. were isolated from the samples collected during the spring (Table-4). Finally, 10 strains were harvested from fishes caught during the autumn and 4 strains were collected during the winter. This seasonal trend observed may be related to the increase in temperature and salinity of seawater during the summer season, stimulating *Vibrio* growth. The limited recovery of *Vibrio* spp. in winter is related to their ability to remain quiescent, in a viable but non-culturable state, while retaining their virulence [50].

Considering the sampling area, the largest number (n=26) of strains was isolated from cultured fishes in the Boumerdes area (F1 and F2), while 16 strains were isolated from the farmed fishes sampled in the Tipaza area (F4). No *Vibrio* spp. occurred in the farm location F3 situated in the Azefoune area. The prevalence and distribution of the strains may be related to the farm setting and may also be related to the degree of seawater contamination. The presence of industrial plants and certain discharges in proximity to the farms may lead to favorable conditions for *Vibrio* growth, especially during the summer months when seawater is warmer. Therefore, the high level of contamination of *Vibrio* found in this area may also explain the detection of *V. alginolyticus* and *V. cholerae* strains during the winter season when, usually, the isolation of cultivable forms is rare [28,32,51]. From the Boumerdes area, 17 strains were isolated from farm F1, while 9 strains were isolated from farm F2. Interesting to note is that both these two farms receive wastewater from neighboring industrial plants and differ only in their specific farm setup. The land-based setting of F1 may be related to the greater number of isolated samples than found in F2, having a positive impact on *Vibrio* growth therein. Instead, no *Vibrio* strains were isolated from fishes caught in Azefoune.

The incidence rate of *Vibrio* spp. considering all the 690 fishes analyzed was 6.08% while, among the 530 cultured fishes, the percentage increased up to 7.92%. The results obtained are consistent with a similar study conducted in Morocco on 220 samples of fishery products, where 8.2% of *Vibrio* spp. incidence was detected [14]. However, our data are at odds with the sole study performed on different seafood collected in Algeria, in which only one strain was isolated out of 200 fish samples [49]. The high reported prevalence (2.83%) of *V. cholerae* observed in the farmed fishes in our study represents a pressing concern, considering the potentially high pathogenicity of this strain responsible for important foodborne outbreaks [52,53].

In addition, further attention should be paid to *V. alginolyticus*, an opportunistic pathogen in gilt head sea bream and humans [54-56], and to *V. fluvialis*, which is considered an important emerging pathogen[17]. All these pathogens could infect fishes subject to stress factors such as breeding and catching associated with predisposed factors like the increase in water temperature during the summer season. In this regard, some pathogens of *Vibrio* spp. are able to contaminate edible portions of fish directly during its conservation or indirectly in the handling of fish products [55,57,58]. Given the wide consumption of fishery products in Algeria and the low number of literature data on *Vibrio* spp. as pathogenic agents in Algeria and beyond, it is clearly important to understand their distribution in marine environments as well as to develop methodologies for detecting viable but non-culturable forms of these organisms [59].

## Conclusion

The present work advances substantially, with a high number of samples (n=690), the lacking and dated studies on the presence of pathogenic *Vibrio* along the Algerian Mediterranean coast in wild and farmed *S. aurata* and *D. labrax*. In fact, surveying, monitoring, and detecting pathogens in foods are the most crucial approaches to reduce, control, or prevent foodborne pathogenic diseases [60]. The reported incidence of *Vibrio* and the presence of potentially pathogenic strains such as *V. cholerae* raise particular concern among Algerian farmed fish consumption, especially considering the possibility of cross-contamination in food production and the increasing consumption of raw fish products (sushi and sashimi) and marinated fish products [61]. The respect of storing temperature (0-2°C), the application of preservation techniques [62], alongside the use of decontaminating agents such as bacteriophages and natural compounds [63-65], could reduce the risks related to the consumption of these fish products. However, considering the growing expansion of Algerian fish farms production and the tangential increase in the number of consumers exposed to potentially pathogenic *Vibrio* spp., the improvement and monitoring of hygienic farm conditions are highly necessary for food safety improvements.

## Authors’ Contributions

SA and AB conceived the study designed. SA performed the experiment. FG and LN analyzed the data. FG and LN drafted and revised the manuscript. All authors read and approved the final manuscript.
